# The Ubiquitin-Conjugating System: Multiple Roles in Viral Replication and Infection

**DOI:** 10.3390/cells3020386

**Published:** 2014-05-06

**Authors:** Arianna Calistri, Denis Munegato, Ilaria Carli, Cristina Parolin, Giorgio Palù

**Affiliations:** Department of Molecular Medicine, University of Padova, via Gabelli 63, Padova 35121, Italy; E-Mails: denis.munegato@studenti.unipd.it (D.M.); ilaria.carli@unipd.it (I.C.); cristina.parolin@unipd.it (C.P.); giorgio.palu@unipd.it (G.P.)

**Keywords:** ubiquitin, post-translational modification, viruses, human immunodeficiency virus

## Abstract

Through the combined action of ubiquitinating and deubiquitinating enzymes, conjugation of ubiquitin to a target protein acts as a reversible post-translational modification functionally similar to phosphorylation. Indeed, ubiquitination is more and more recognized as a central process for the fine regulation of many cellular pathways. Due to their nature as obligate intracellular parasites, viruses rely on the most conserved host cell machineries for their own replication. Thus, it is not surprising that members from almost every viral family are challenged by ubiquitin mediated mechanisms in different steps of their life cycle and have evolved in order to by-pass or exploit the cellular ubiquitin conjugating system to maximize their chance to establish a successful infection. In this review we will present several examples of the complex interplay that links viruses and the ubiquitin conjugation machinery, with a special focus on the mechanisms evolved by the human immunodeficiency virus to escape from cellular restriction factors and to exit from infected cells.

## 1. Introduction

Ubiquitin (Ub) is a highly conserved protein of 76 aminoacids that can be covalently linked to target proteins through a multistep process known as ubiquitination. Protein ubiquitination represents one of the best characterized post-translational modifications that controls the fate/function of proteins [1]. Protein ubiquitination involves a series of cellular enzymes in an enzymatic cascade, starting with the Ub-activating enzyme E1, followed by the ubiquitin-conjugating enzyme E2 and by the Ub ligase E3, which form an isopeptide bond between the carboxyl terminus of Ub and the ε-amino group of a lysine residue on the target protein [2]. The E3 ligase usually determines the substrate specificity, although the E2-conjugating enzyme can also play a role in the substrate selection. Accordingly, while there are few known E1 enzymes in mammals and roughly thirty five E2s in humans, hundreds of E3/Ub ligases have been identified so far. E3 enzymes are currently classified into three main classes with different structural and functional characteristics: the HECT domain family of Ub ligases, the Cullin-RING family of Ub ligases, and the U-box containing Ub ligases [3,4,5]. 

**Figure 1:**
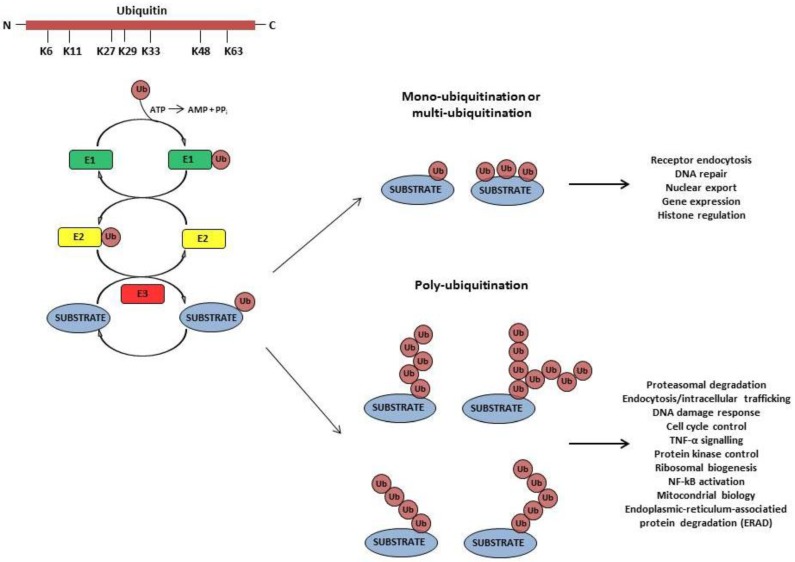
Schematic representation of the Ub molecule and of the enzymatic cascade leading to protein ubiquitination. The seven lysines (K) involved in the process, the ubiquitin-activating enzyme E1, the ubiquitin-conjugating enzyme E2 and the ubiquitin ligase enzyme E3 are highlighted, along with the main fate of the target proteins.

The final outcome of the first round of the ubiquitination cascade is the mono-ubiquitination of the target protein. After mono-ubiquitination, a specific lysine of the first Ub can be used by the same set of proteins to mediate the consecutive attachment of additional Ubs, resulting in the formation of poly-Ub chains (Figure 1). In the most studied event, the ubiquitinated misfolded or damaged cytoplasmic and nuclear proteins are delivered to the proteasome for degradation as final event of the well-known Ub-proteasome system (UPS) [6]. On the other hand, ubiquitination of the cytoplasmic domains of transmembrane proteins results in their sorting to lysosomes via the multivesicular body (MVB) pathway [7]. Ub-mediated degradation is important not only for the regulation of protein turn-over, but it also plays a role in DNA damage repair, cell-cycle regulation, cellular growth, as well as in the immune system functions [8]. 

Moreover, it has been demonstrated that Ub can also function act independently from its proteolytic activity, by regulating protein function and protein/protein interaction [8,9]. An important role in this context is played by specific Ub hydrolases (deubiquitinating enzymes or DUBs) that catalyze the removal of Ub from the target proteins. Similar to the function of kinases and phosphatases during the phosphorylation process, Ub ligases and DUBs can affect substrate function by transient ubiquitination. Thus, protein ubiquitination represents a highly versatile and reversible event that may influence different features of a protein and not only its stability. Part of this versatility is clearly linked to the fact that Ub contains at least seven lysines (K) and additional residues that can be employed by the Ub ligases to generate different types of Ub chains on the target proteins, which, in turn, will interact with different downstream factors (Figure 1) [1]. For instance, it is well established that K-48-based linkages lead mainly to the proteasome-mediated degradation of the ubiquitinated protein, while K-63-based Ub chains control primarily protein endocytosis, as well as trafficking and enzyme activity (Figure 1) [10,11]. 

In addition to Ub, a number of Ub-like (UbL) proteins can also be conjugated to target substrates by specific E1, E2, and E3 enzyme-like proteins. Among them are SUMO (Small Ub modifier), ISG15 (Interferon-stimulated gene 15), NEDD8 (Neural precursor cell expressed, developmentally down-regulated 8), FAT10 (HLA-F adjacent transcript 10), Atg12 (Autophagy-related protein 12) and LC3 (microtubule-associated protein 1 light chain 3) which display different functions and roles in the cellular physiology [12].

As obligate intracellular parasites with limited genome size, viruses must co-opt the host cellular machineries in almost every step of their life cycle, from entry into the host cell, replication and transcription of their genome (either RNA or DNA), synthesis of proteins, assembly of new particles, till the egress from the infected cell. In addition, in order to establish an infection, viruses must overcome different host immune defenses. Thus, there is a constant interaction between the virus and its host, with the virus trying to either counteract or exploit different complex cellular mechanisms and pathways to efficiently produce infectious progenies, or to establish a long-term persistence in the host, depending on the virus taken into consideration. Under this respect, given the role played in many cellular fundamental processes, it is to be expected that Ub and UbL proteins are involved in almost every aspect of the viral life cycle and pathogenesis [13]. 

In this review, we will present different examples of how viruses interact with Ub/UbL-mediated cellular processes in order to optimize their chance of survival, with a special focus on the mechanisms evolved, in this context, by one of the most important human pathogens, the Human Immunodeficiency Virus type 1 (HIV-1). 

## 2. Viruses Employ the Ub-Conjugating System to Accomplish Different Steps of Their Replication and to Establish a Successful Infection

The first evidence supporting the ability of viruses to utilize the UPS to their own advantage came from the study of small DNA tumor viruses and of their capability to interfere with the regulation of the cell cycle [14]. Since then, it has become clear that members of almost all viral families subvert or exploit both the cellular Ub-conjugating and -deconjugating machineries in different phases of their replication cycle [15,16,17,18,19,20,21,22,23,24,25,26,27,28]. Under this respect, studies based on the treatment of infected cells with proteasome inhibitors have been instrumental, as such a treatment not only blocks the UPS, but also depletes the cellular pool of free Ub, affecting *de facto* all the cellular pathways involving this protein. Proteasome inhibitors have been shown to interfere with the replication of major human pathogens such as herpesviruses [15,16], poxviruses [17,18], hepadnaviruses [19], adenoviruses [20], influenzaviruses [21], retroviruses [22,23,24], coronaviruses [25], paramyxoviruses [26], picornaviruses [27] and rotaviruses [28].

Ub already plays a role in the first steps of viral replication. As an example, proteasome inhibitors have been reported to inhibit herpes simplex virus (HSV) entry at an early step, immediately after the penetration of the viral capsid into the target cell [15,29]. The Kaposi Sarcoma associated herpesvirus (KSHV) [30], the influenza virus [31] and adenoviruses [32] are additional examples of viruses which rely upon the UPS for their entry into target cells. For instance, it has been demonstrated that the UPS is linked to the ability of the human pathogen KSHV to penetrate into endothelial cells and to traffic to the nuclei. 

Not only entry and early post-entry events, but also other steps of the viral life cycle can be affected by impairment of the proteasome activity. Indeed, Ub-mediated mechanisms regulate gene expression in Epstein-Barr virus (EBV) [33], HIV-1 [34], and human T lymphotropic virus (HTLV) [35]. In all these cases, specific viral proteins which function as transcriptional transactivators are able to interact with the Ub/UbL-conjugating machinery, leading to an increase in viral protein activity.

UPS is also involved in the ability of herpesviruses to establish lifelong infections in their hosts, a phenomenon known as latency, as reported for KSHV [36,37] and EBV [38,39]. Specifically, in the case of EBV, the viral latent membrane protein 2A (LAMP2A) [38] and LMP1 [39] regulate viral lytic/latent replication through the interaction with specific Ubligases and DUBs. 

Finally, Ub plays a role in viral release from infected cells [40], as it will be discussed in more details later on. 

## 3. Viruses Usurp the Ub-Conjugating System to Evade Host Immune Responses

Innate immunity represents a first line of defense employed by the cells against microorganisms. Microorganisms are recognized by specific molecules named pattern recognition receptors (PRR) that bind to pathogen-associated molecular patterns (PAMPs). This binding leads to the activation of different signaling cascades, with the involvement of the pro-inflammatory transcription factors AP-1, NF-kB and/or one or more members of the interferon-regulatory factor (IRF) family, with the final production of pro-inflammatory cytokines and interferon (IFN). Not only regulation of innate immune signaling relies on post-translational modifications such as conjugation of Ub/UbLs to keys cellular proteins [41], but the Ub/UbL conjugating system itself is adopted by many viruses to counteract this host defense response [42]. 

First of all, viruses prevent the induction of NF-kB and/or IFN. To this end, the use of the cellular Ub conjugating machinery represents one of the most exploited strategy. NF-kB is a complex of dimeric transcription factors, which in mammals comprises RelA (p65), RelB, c-Rel, NF-kB1 (p50) and NF-kB2 (p52) [43]. In normal conditions, NF-kB dimers are bound to NF-kB inhibitory proteins (IKBs) and retained in the cytoplasm. Upon activativation, a multiprotein complex constituted by the enzyme transforming growth factor beta activated kinase-1 (TAK1), the NF-kB essential modifier (NEMO), and the IkB kinase (IKK) is formed. This complex phosphorylates the NFkB inhibitor (IkB), leading to its ubiquitination and degradation by the proteasome. Once released by IkB, NF-kB can translocate into the nucleus where, along with specific IRFs and other co-factors, is able to stimulate IFN transcription. Among other mechanisms, it has been described that viruses can directly influence NF-kB stability. For instance, the case of the murid herpesvirus-4 (MuHV-4) latency associated protein ORF73 leads to p65/RelA degradation. [44]. Viruses can also use UPS to impair NF-kB translocation to the nucleus by blocking IkB degradation. For instance, the rotavirus NSP1 protein mediates the ubiquitination and degradation of the β-transducin repeat containing protein (β-TrCP) that, otherwise, would bind to and degrade IkB [45]. Viruses can also affect the stability of IRFs and in particular of IRF3. One example is given by the Varicella Zoster Virus (VZV) ORF61, a protein displaying a RING finger E3 ubiquitin ligase activity. ORF61 specifically interacts with the phosphorylated/activated form of IRF3 leading to its ubiquitination and proteasome mediated degradation [46]. 

In addition, viruses can act downstream the IFN induction, by inhibiting the signal cascade activated by IFN binding to its receptor [47]. As an example of UPS-mediated viral interference with proteins belonging to this cascade, viruses in the Rubulavirus genus of the *Paramyxoviridae* family lead STAT proteins to proteasome-mediated degradation, by assembling STAT-specific ubiquitin ligase complexes from cellular components [48].

Finally, viruses can directly target the antiviral IFN-induced proteins (ISGs) [49]. Some of these proteins, such as the Protein Kinase R (PKR), the 2’,5’-oligoadenylate-synthetase (OAS), the ribonuclease L(RNAseL), and the Mx GTP-ase, along with the respective viral counteracting mechanisms have been extensively studied [49,50,51]. Recently, different reports have been focused on the ISGs belonging to the Tripartite motif (TRIM) containing family, especially after the discovery of the role played by TRIM5α in the establishment of a cross-species barrier against HIV-1 infection [52]. Interestingly, TRIM5α and many other members of this family of ISGs function by ubiquitinating, SUMOylating or ISGylating host/viral proteins with different outcomes on the antiviral response [53,54]. Not only, the ISG15 Ub-like protein is itself an ISG, and one of the most potently induced upon viral infection. It has been demonstrated that ISG15 displays a broad antiviral activity [55], even though the mechanism/s accounting for this effect is/are still under investigation. What is known is that ISGylation can specifically inhibit the functions of an Ub ligase, Nedd4, which plays a role in the replication cycle of different RNA viruses, such as Ebola virus and oncoretroviruses [56]. Moreover, it has been reported that viruses have evolved the ability to remove ISG15 from target proteins. For instance, the coronavirus papain-like protease (PLP) protein acts as a de-ubiquitinating and de-ISGylating enzyme [57]. Another interesting ISG is represented by a peculiar cellular protein, tetherin, that being one of the most recently characterized targets of the HIV-1 accessory protein Vpu, will be described in more details later.

## 4. Viruses have Evolved Different Strategies to Exploit the Ub-Conjugating System

This rapid overview on the involvement of UPS in crucial steps of the viral life cycle suggests immediately that viruses are connected to Ub in different ways, either by usurping the host’s Ub-conjugating system or by evolving their own one. 

### 4.1. Viruses Subvert the Cellular Ub-Conjugating System

First of all, viral proteins have been described that can modify the substrate specificity of cellular Ub ligases. As a consequence, specific cellular proteins are targeted for degradation. Such a strategy, is exploited by viruses in several of the examples described in the previous paragraphs. For instance, the MuHV-4 ORF73 represents one of the viral proteins that can subvert the substrate recognition of a cellular E3 enzyme. Indeed, by binding to ORF73, the ElonginC/Cullin5/SOCS Ub-ligase complex is able to poly-ubiquitinate p65/RelA with its subsequent proteasomal degradation [44]. The rotavirus NSP1 protein leads to β-TrCP ubiquitination and degradation through the recruitment of the ubiquitin-ligase complex Skp-1/Cul1/F-Box (SCF) [45]. Furthermore, small DNA viruses with known oncogenic activity, such as the human papillomavirus (HPV), adenoviruses and polyomaviruses, take control of the cell cycle by usurping specific cellular Ub ligase complexes to target crucial cell cycle regulators such as p53 and the protein of the retinoblastoma (pRB) for degradation [58]. In this way, two of the best studied tumor suppressor cellular pathways are inactivated. Indeed, different types of HPV (high-risk) are strongly linked to the onset of cervical cancers, as well as to other type of cancer such as those affecting vagina, vulva, penis, and the head and neck [59], while many others, classified as low-risk, are only very rarely associated with cancer. Although the high-risk and low-risk HPVs share several biological features, the two groups display significant structural/functional differences at the level of the two main viral encoded oncogenes: E6 and E7. Indeed, high- and low-risk E6 and E7 proteins have a different ability in affecting p53/pRB stability and in modulating the activity of additional cellular proteins with an effect on cell cycle control and cellular proliferation [59]. Interestingly, the studies of viral interactions with the host cell cycle have been instrumental in the identification and characterization of p53 and pRB [60]. 

In addition to p53 and pRB, another protein complex involved in cell cycle control, the anaphase-promoting complex (APC) is emerging as a key target for viral proteins. APC is a cullin-RING E3 Ub ligase that leads to the proteasome degradation of multiple cell cycle regulators and, as a consequence, to the correct progression of the cell cycle itself [61]. Different viruses have been reported to affect the APC function. In particular, the human cytomegalovirus (HCMV), a known human pathogen, encodes a protein, pUL21a, that is able to induce proteasome-dependent degradation of APC subunits during viral infection [62]. Additional viruses, and among them important human pathogens with known oncogenic activity, such as the HTLV-1, HPV, hepatitis B virus (HBV) have been reported to interfere with APC [61]. 

Viral proteins themselves can be directly modified by Ub or Ub-like proteins and, as a consequence, they can be recognized by cellular pathways that can be then exploited by the virus to perform specific tasks. Examples of these processes are found in the mechanisms evolved by several enveloped viruses to egress from infected cells, as it will be explained later.

Viruses can also alter the activity of cellular de-ubiquitinating enzymes, by encoding, for instance, proteins which are able to interact with cellular DUBs. Under this respect, the EBV EBNA1 protein, which is involved in several crucial aspects of viral replication and pathogenesis such as maintenance of the viral genome, transcription and translation of the viral DNA, viral persistence, and cellular transformation, interacts with USP7 or herpesvirus-associated Ub-specific protease (HAUSP) [63,64,65]. This cellular DUB is able to remove Ub from p53 and from EBNA1 itself, thus preventing their degradation [66]. During EBV infection, not only USP7 but several additional cellular DUBs are known to increase their activity and one of the effects appears to be the stabilization of β-catenin, a key factor of the Wnt signaling pathway. The disregulation of this signaling pathway has been implicated in tumor development [67]. Since EBV is associated, as well, with different types of cancers, such as Burkitt’s and Hodgkin’s and the nasopharyngeal carcinoma, the study of EBV interference with the Wnt pathway and the role played by the cellular DUBs in this context are relevant.

### 4.2. Viruses have Evolved Their Own Ub-Conjugating System

Some viruses, especially the large DNA viruses such as herpeviruses and poxviruses, encode their own ubiquitinating enzymes (Table 1). VZV ORF61 mentioned above is an example of such viral encoded Ub ligases. Additional examples are found in the processes evolved by different viruses to overcome the host immune defenses. Under this respect, KSHV encodes two E3 Ub ligases, K3 and K5, able to ubiquitinate the class I major histocompatibility complex (MHC), leading to its downregulation from the cell surface or from the ER and thus interfering with cell antigen presentation [68]. K5 also affects other surface proteins important for the stimulation of T cells, such as ICAM-1 and B7-2 [69]. Interestingly, several other members of the *Herpeviridae* family have been described to down-regulate MHCI and T-cell activation markers from the cell surface by employing different mechanisms [70], some relying again on Ub, as in the case of HCMV [71]. Another interesting example is given by the HSV-1 ICP0 protein, a multifunctional factor which displays, among other features, a RING domain E3 Ub ligase activity [72]. Thanks to this activity, ICP0 is able to disassemble cellular protein aggregates, known as promyelocytic leukemia nuclear bodies (PML NBs), that are present in the nucleus of infected cells where they are involved in different processes such as the interferon (IFN) response to viral infection [73]. Both herpesviruses and adenoviruses have been described to interfere with PML NBs [73] and UPS is at the basis of some of the mechanisms employed by these viruses to this end. HSV-1 ICP0, in particular, mediates the proteasomal degradation of one of the protein complexes that constitute these bodies [74]. 

**Table 1:** A list of the viral proteins with Ub ligase activity along with their characterized substrates are reported.

Virus	Viral protein	Target protein	Reference
Herpes Simplex Virus type 1	ICP0	pUL46 (viral)	Lin *et al*. 2013 [75]
		p65, p50	Zhang *et al*. 2013 [76]
		CENPs	Gross *et al*. 2012 [77]
		microtubule	Liu *et al*. 2010 [78]
		RNF8,RNF168	Lilley *et al*. 2010 [79]
		PML	Isaacson *et al*. 2009 [80]
		Sp100	
		Cyclin D3	
		p53	
		USP7	
		ICP0 (viral)	
Kaposi Sarcoma-associated herpesvirus	K3	MHC-I	Timms *et al*. 2013 [81] Isaacson *et al*. 2009 [80]
		CD1d	Boname *et al*. 2011 [68]
		PECAM	
		IFN-γ R1	
	K5	MHC-I	Boname *et al*. 2011 [68]
		Tetherin/BST-2	Boname *et al*. 2011 [68] Pardieu *et al*. 2010 [82]
		ICAM-1	Timms *et al*. 2013 [81] Isaacson *et al*. 2009 [80] Boname *et al*. 2011 [68]
		B7-2	
		CD1d	
		HFE	
		PECAM	
		ALCAM	
		MIC-A/-B	
		AICL	
		DC-SIGN DC-SIGNR	Lang *et al*. 2013 [83]
Kaposi Sarcoma-associated herpesvirus		AICL	Boname *et al*. 2011 [68]
		VE-Cadherin	
		IFN-γ R1	
		Syntaxin-4	
		BMPRII	
		RTKs	Karki *et al*. 2011 [84]
Varicella Zoster Virus	ORF61p	ORF61p (viral)	Walters *et al*. 2010 [85]
		IRF3	Zhu *et al*. 2011 [46]
Adenovirus	E1B-55k,E4orf6	p53	Woo *et al*. 2007 [86]
		MRN complex	
Murine gamma herpesvirus 68	ORF75c	PML	Sewatanon *et al*. 2013 [87]
Rodent herpesvirus Peru	pK3	pK3 (viral)/MHC-1	Herr *et al*. 2012 [88]
		MHC-I membrane bound chaperons	
Poxvirus	p28	unknown	Huang *et al*. 2004 [89]
White Spot Syndrome Virus	WSSV222	TSL	He *et al*. 2009 [90]
Nairovirus	Polymerase	RIG-I	van Kasteren *et al*. 2012 [91]
Murine Hepatitis Virus A59	nsp3	TBK1	Wang *et al*. 2011 [92]
		IRF3	Zheng *et al*. 2008 [93]
Foot-and-mouth Disease Virus	L(pro)	RIG-I	Wang *et al*. 2011 [94]
		TBK1	
		TRAF6	
		TRAF3	
Hepatitis B Virus	HBx	RIG-I	Jiang *et al*. 2010 [95]
		TRAF3	

Moreover, viral DUBs have been described (Table 2). Among them, the large tegument protein of herpesviruses belonging to all the three known families of these pathogens (α, β and γ) not only is an essential component of the viral particle, but displays a conserved and unique deubiquitinating activity [96]. Taking into account the functions played by the large tegument proteins in the herpesviral replication cycle, a role for these viral DUBs during both the entry and the egress of the virus from infected cells is very likely. For instance, the EBV DUB, BPLF1, is known to deubiquitinate and downregulate the viral ribonucleotide reductase [97], the cellular processivity factor PCNA [98] and the E3 ubiquitin ligase Rad18 with a positive effect on the production of infectious particles [99]. It is interesting to note that the herpes simplex virus 1 Ub-specific protease, UL36, has been recently reported to inhibit β-interferon production by deubiquitinating the TNF receptor associated factor 3 (TRAF3) [100]. This finding would indicate a role for viral DUBs in overcoming the cellular immune response, as in the case of several viral Ub ligases. 

**Table 2:** A list of viral proteins with recognized deubiquitinating enzyme (DUB) activity is reported along with the characterized substrates.

Virus	Viral protein	Target protein	Reference
Herpes simplex virus type 1	UL36	TRAF3	Wang *et al*. 2013 [100]
		UL36 (viral)	Bolstad *et al*. 2011 [101]
Human cytomegalovirus	UL48	unknown	Kim *et al*. 2009 [102]
PseudoRabies Virus	UL36	unknown	Bottcher *et al*. 2008 [103]
Kaposi Sarcoma-associated herpesvirus	ORF64	RIG-I	Inn *et al*. 2011 [104]
	RTA	IRF-7	Isaacson *et al*. 2009 [80]
Epstein-Barr Virus	BPLF1	EBV ribonucleotide reductase (viral)	Whitehurst *et al*. 2009 [97]
		PCNA	Kumar *et al*. 2014 [99]
		Rad18	
Crimean-Congo Hemorrhagic Fever Virus	vOTU	Unknown	Akutsu *et al*. 2011 [105]
Marek's Disease Virus	UL36	Unknown	Isaacson *et al*. 2009 [80]
Human coronavirus	PLpro	Unknown	Mielech *et al*. 2014 [106]
Turnip Yellow Mosaic Virus	PRO	Unknown	Lombardi *et al*. 2013 [107]
	98K	RdRp (viral)	Chenon *et al*. 2012 [108]
Porcine Epidemic Diarrhea Virus	PLP2	RIG-I	Xing *et al*. 2013 [109]
		STING	
Porcine Reproductive and Respiratory Syndrome Virus	nsp2	IkBα	Sun *et al*. 2010 [110]
Adenovirus	Avp	Adenoviral and cellular proteins unknown	Balakirev *et al*. 2002 [111]

## 5. Vif, Vpu and Vpr: Three HIV-1 Accessory Proteins that Exploit Cullin-RING Finger Ub Ligase Complexes to Overcome Different Restriction Factors

The impact played by the Ub/UbL system on viral replication and on the establishment of a successful infection is particularly clear when the life cycle and the pathogenetic mechanisms evolved by one of the most studied human pathogens, the HIV-1, is analyzed. HIV-1 is a lentivirus and, like the other members of the *Retroviridae* family, is characterized by a non-icosahedral particle enwrapped in a lipidic envelope. Its genome comprises two copies of single-stranded RNA with a short dimerized region. HIV-1 is the etiological agent of the acquired immunodeficiency syndrome, AIDS, a condition that after 30 years from its discovery and the introduction of the highly active antiretroviral therapy (HAART), still represents one of the major public health problem world-wide [112]. 

**Figure 2:**
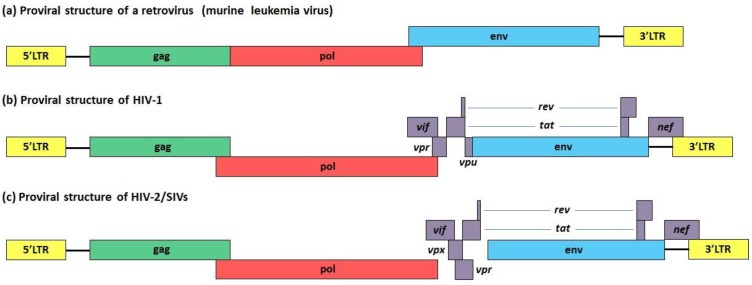
(**a**) The common genetic elements characterizing the proviral DNA of a retrovirus (*gag*, *pol* and *env*) are reported, along with the LTR. Schematic representation of the (**b**) HIV-1 and (**c**) HIV-2/SIV proviral DNAs are also highlighted along with the respective accessory genes.

The HIV-1 life cycle is a typical retroviral replication cycle starting with viral entry into specific target cells, reverse transcription of the viral RNA into double stranded proviral DNA, integration of this proviral genome into the host chromosomal DNA, transcription and translation of viral proteins, assembly of viral particles, followed by their budding from the cell surface with the acquisition of an envelope. All retroviral genomes consist of at least 3 genes, *gag*, *pol* and *env*. In addition to these three main genes, complex retroviruses such as HIV-1 encode accessory proteins that enhance their replication and infectivity (Figure 2). In particular, HIV-1 is characterized by six auxiliary genes (*tat*, *rev*, *nef*, *vpr*, *vpu* and *vif*), of which only two, *tat* and *rev*, are essential for viral replication *in vivo* and *in vitro* [113]. On the other hand, *nef*, *vpu*, *vif*, *vpr* genes encode factors that are known as accessory proteins, as they appear to be dispensable for viral replication in several *in vitro* experimental settings. However, the high degree of conservation of these proteins suggest crucial functions *in vivo*. 

The relevance of HIV-1 as human pathogen and the consequent development and availability of different tools and techniques to manipulate its genome, has allowed to deeply dissect several aspects of HIV biology. Even though different questions still need to be answered, the reliance of HIV-1 on numerous cellular pathways and factors for nearly every step of its replication is well appreciated [114,115,116]. Moreover, it is becoming clear that HIV-1 proteins, and especially the accessory proteins, have the ability to antagonize host molecules that represent first lines of defense against retroviral infections. These cellular proteins are known as intrinsic immunity factors or restriction factors [117]. Recent studies have highlighted how the HIV-1 accessory proteins Nef, Vif, Vpu, and Vpr have evolved in order to enable the virus to evade the host immune system [118]. Interestingly, a common mechanism of action of these proteins is the use of the UPS to interfere with cellular proteins that would affect HIV-1 replication. In particular, Vif, Vpu, and Vpr exploit and subvert the physiological activity of specific cullin-RING finger Ub ligases (CRLs) to induce the polyubiquitination and proteasomal degradation of specific cellular targets [119,120] (Table 3). CRLs are the largest family of Ub ligases and are responsible for ubiquitination of almost 20% of cellular proteins degraded through the UPS. The choice as target of members of this particular family of E3 enzymes operated by these HIV-1 proteins is not accidental. Indeed, CRLs are multisubunit complexes composed of a cullin (at least seven cullins are known in vertebrates), an Rbx/Roc RING finger protein, a variable substrate-recognition subunit (SRS), and in most cases, an adaptor that links the SRS to the complex [4,121,122]. Thus, CRLs are extremely versatile comprising hundreds of distinct complexes with the potential to recruit several protein. This feature has been exploited by Vif, Vpu and Vpr to optimize the chances of HIV-1 to overcome different restriction factors evolved by the cells to inhibit viral replication and spreading. 

**Table 3:** Cullin-RING finger Ub ligase complex usurped by the HIV-1 Vif, Vpu and Vpr accessory proteins with the respective target proteins and biological effects that have been identified so far.

Viral protein	Cullin-RING finger Ub ligase complex	Target protein	Biological effects	References
Vif (HIV-1)	CBF-β-ElonginB-ElonginC-Cullin5-Rbx	APOBEC3 (A3)	Prevention of A3s incorporation into the budding virions Prevention of proviral DNA hypermutation	Guo *et al*. 2014 [123]
Vpu (HIV-1)	Skp1-Cullin1-F box	CD4	Retention in the ER and delivery to the ER-associated degradation (ERAD) pathway Prevention of superinfection	Nomaguchi *et al*. 2008 [124]
		BST2/Tetherin	Promotion of viral egress	Goffinet *et al*. 2009 [125] Mangeat *et al*. 2009 [126] Douglas *et al*. 2009 [127]
		p53	Stabilization of p53 and enhancement of apoptosis	Verma *et al*. 2011 [128]
		Ubiquitination of Vpu	Unknown (stabilization or proteasomal degradation)	Belaïdouni *et al*. 2007 [129]
Vpr (HIV-1)	Cullin4A-DDB1-DCAF1 Cullin4B also involved (Sharifi *et al*. 2014)	Unknown cellular substrate(s)	G2 cell cycle arrest	Le Rouzic *et al*. 2007 [130]
		UNG2 and SMUG1	Unknown	Eldin *et al*. 2014 [131]
		Dicer	Suppression of RNA silencing pathway	Casey Klockow *et al*. 2013 [132]
Vpx (HIV-2, SIV)	Cullin4A-DDB1-DCAF1 Cullin 4B also involved (Sharifi *et al*. 2014)	SAMHD1	Increase of the intacellular pool of dNTPs Efficient synthesis of viral DNA	Sze *et al*. 2013 [133]

### 5.1. Vif and APOBEC Proteins

Vif is a 23KDa protein that is required for production of infectious virus in a cell type-specific manner [134]. Experimental evidence indicated that cells non permissive to *vif*-defective HIV-1 express a host factor inhibiting viral replication [135], next identified in the cytidine deaminase APOBEC3G [136]. APOBEC3G is clearly expressed in *vif*-defective HIV-1 nonpermissive cells where it acts as an intrinsic restriction factor. In the absence of Vif, APOBEC3G is incorporated into budding virions, and, in the newly infected cells, it determines cytidine to uracil mutations in the single stranded DNA of HIV-1, during the process of reverse transcription. This hypermutation results in viral replication impairment. When expressed, Vif recruits a multi-subunit E3 Ub ligase complex composed of a scaffold protein, Cullin 5, RING-box protein, a SOCS box binding protein complex, Elongins B/C, as well as the core binding factor beta (CBF-β) [137]. Vif directly binds Cullin 5 [137]. The Vif-CBF-β-ElonginB-ElonginC-Cullin5-Rbx E3 complex is then able to polyubiquitinate APOBEC3G leading to its protesomal degradation [138]. As a consequence, APOBEC3G is not incorporated into viral particle and HIV-1 can replicate in *de novo* infected cells. It has to be mentioned that additional APOBEC molecules (i.e. APOBEC3DE/3F) have been characterized for their ability to restrict *vif*-defective HIV-1. In Vif expressing cells, all these restriction factors are substrates of the same E3 complex described in the case of ABOBEC3G and are as well subjected to proteasomal degradation [139]. Interestingly the crystal structure of the Vif/CRL complex has been recently resolved [123]. This finding will help to further clarify the molecular basis of Vif function. 

### 5.2. Vpu and Tetherin

Vpu is an 81 amino acid dimeric integral membrane protein. One of the first characterized functions of this HIV-1 accessory protein was the ability of recruiting a CRL (Skp1-Cullin1 E3 ligase complex) to the cytoplasmic tail of CD4, inducing its downregulation at the level of the ER [124]. In particular, Vpu acts as an adaptor protein, directly interacting with CD4 in the ER and with β-TrCP, a component of the Skp1-Cullin-F box (SCF) ubiquitin ligase complex, leading to the CD4 polyubiquitination, dislocation from the ER to the cytosol and proteasomal degradation [124]. In addition to this important function, which is likely required for proper trafficking and maturation of the viral envelope glycoproteins, Vpu has been more recently characterized for yet another crucial role, connected with the ability of the virus to evade a specific IFN-1 induced antiviral factor: the B cell stromal factor 2 (BST-2) or tetherin. The latter name perfectly reflects the activity of BST-2 that, in the absence of Vpu, physically retains (tethers) fully assembled HIV-1 particles to the surface of the infected cells [140]. Indeed, BST-2 is a type II transmembrane protein with an unusual topology consisting of an N-terminal cytoplasmic tail, a transmembrane domain, a coiled-coil extracellular domain, and a glycosylphosphatidylinositol anchor at the carboxy-terminus. It has been demonstrated that this peculiar conformation, rather than the primary sequence, confers to tetherin the ability to physically retain budding virions on the cellular membrane [141]. HIV-1 is not the only virus sensitive to BST-2 [142] and human cells are not the only one encoding for such a restriction factor [142,143,144,145]. Indeed, tetherin represents also one of the major cross-species barrier factor especially in the context of lentiviral infection [143,144,146,147,148]. 

There is still some uncertainty as to how Vpu antagonism is accomplished, as different mechanisms have been reported. Indeed, it has been described that Vpu directly interacts with tetherin and can mediate its down-regulation from the cell surface with an increase in viral release [125]. While some studies have indicated a Vpu mediated β-TrCP-dependent proteasomal degradation of tetherin [125,126], there are also data supporting a role for the β-TrCP-dependent endo-lysosomal pathway in BST-2 degradation [127,149]. In agreement with these latter studies Rab7A, a small GTPase essential for the maturation of late endosomes and lysosomal fusion, appears to be required for tetherin degradation [150], as along with the Endosomal Sorting Complex Required for Transport (ESCRT)-0 [151]. Interestingly, β-TrCP2-dependent ubiquitination and subsequent degradation of tetherin do not seem to require lysine residues in the cytoplasmic domain of tetherin [152]. It has to be mentioned that other studies have revealed a β-TrCP2-independent mechanism of tetherin antagonism by Vpu, with the delocalization and retention of tetherin in a perinuclear compartment in the absence of degradation [153,154]. Even though degradation does not appear to be the primary system by which Vpu counteracts BST-2, sequestration and degradation might be parallel existing and not mutually exclusive mechanisms by which Vpu optimizes the chances to counteract the antiviral effect of tetherin. In agreement with the concept of multiple mechanisms, other viral proteins are able to counteract tetherin function, both by inducing its Ub-dependent endosomal degradation, as in the case of the KHSV K5 protein [82], or by sequestration in a perinuclear compartment, as in the case of the HIV-2 Env [155].

### 5.3. Vpr and Vpx

Vpr is a 96 amino acid protein whose function has been difficult to elucidate. One of the clearest activities of Vpr is its ability to delay or arrest cells in the G2 phase of the cell cycle. In addition, in this case, the recruitment of a specific CRL appears to be essential. Indeed, Vpr interacts with the cullin4A-DDB1-DCAF1 Ub ligase complex [130,156,157,158,159,160,161,162], a binding that is required for the induction of the G2 arrest [163]. However, the exact target/s of Vpr-cullin4A-DDB1-DCAF1 activity that are linked to the G2 cell-cycle arrest is/are still unknown [164]. In particular, the two substrates of Vpr-mediated degradation, that have been better characterized so far, the uracil-DNA glycosylase (UNG) 2 and the single-strand selective monofunctional uracil-DNA glycosylase (SMUG1) [131] do not seem to account for the above described Vpr effect. Recently, it has been demonstrated that also the endoribonuclease Dicer is subjected to proteosomal degradation via Vpr-recruited cullin4A-DDB1-DCAF1 Ub ligase, with enhanced HIV-2 replication in monocyte-derived macrophages [132]. It is well known that Dicer is involved in the generation of miRNA, a major component of the RNA silencing machinery interfering with viral replication. Thus, Vpr would also act as a suppressor of silencing (SRS) and Vpr-mediated degradation of Dicer would represent another example of a cellular restriction mechanism to HIV-1 infection bypassed by a viral “usurped” CRL complex.

While the Vpr accessory protein is encoded by all primate lentiviruses, including HIV-1 and HIV-2, its paralog Vpx is expressed only by HIV-2 and by certain simian lentiviruses. Myeloid cell types are known to be less permissive to HIV-1 infection with respect to CD4-positive T lymphocytes [133,165]. This difference in susceptibility to HIV-1 infection has been linked to the expression in myeloid cell types of the Vpx-interacting protein SAMHD1 [133]. SAMHD1 is a nucleotide triphosphohydrolase that can inhibit lentiviral reverse transcription by depleting the intracellular pool of available dNTPs [133,166]. The HIV-2 and SIV Vpx proteins counteract this restriction by inducing SAMHD1 degradation following its ubiquitination. Once again, as just described in the case of Vpr, the CRL involved in such a process is the Vpx recruited cullin4A-DDB1-DCAF1 [118,167]. It has been recently reported that HIV Vpr and Vpx exploit not only cullin4A but also cullin4B to mediate ubiquitination of target proteins. Interestingly, in primary macrophages Vpx appears to need both cullin4A and cullin4B to obtain maximal SAMHD1 degradation [168]. Since HIV-1 does not have a Vpx protein and HIV-1 Vpr is not capable of interacting with SAMHD1, myeloid cell types display resistance to HIV-1 infection. Thus, SAMHD1 represents an additional restriction factor that interferes with HIV-1 infection, at least in a specific cell type, and that can be overcome by the virally recruited cullin4A-DDB1-DCAF1 Ub ligase.

## 6. Role of the Ub Conjugation System in Viral Egress from Infected Cells

Viruses have developed sophisticated mechanisms for exiting from infected cells. Enveloped viruses leave the cells through a complex process, known as budding, that requires two main steps, (i) the cell membrane deformation around the assembling virions; and (ii) a fission, resulting in the detachment of the viral particles from the cellular surface [169,170]. 

Studies on retroviruses, and in particular on HIV-1, have been instrumental in dissecting the complex viral/cellular interplay which ensures a successful egress of enveloped viruses. Indeed, the Gag polyprotein is the only retroviral protein necessary at this level. This feature allowed the development of viral mutants and tools to identify the molecular mechanisms involved in budding. In 1991, Göttlinger and co-workers, along with other groups, identified in the C-terminal p6 domain of HIV-1 Gag a highly conserved motif (PT/SAP) as crucial player in the detachment of budded virions from the cell surface [171,172]. Starting from these initial findings, short proline-rich sequences, named late assembly or L-domains, functionally equivalent to the HIV-1 PT/SAP motif, have been identified in the Gag of different retroviruses [173]. To date, in addition to the PT/SAP motif, typical of most lentiviruses, two further L-domains have been well characterized: the PPXY-type L-domain present in the Gag proteins of oncoretroviruses and the YPX_n_L-type motif, identified in the Gag protein of the equine infectious anemia virus (EIAV) [56]. Besides retroviruses, L-domains have been also found in the structural proteins of most RNA enveloped viruses such as rhabdoviruses, filoviruses, arenaviruses, and paramyxoviruses [56], and in some DNA enveloped viruses [174,175,176,177,178]. Several data have indicated a connection between Ub, L-domains and retroviral egress from infected cells. Firstly, a functional L-domain leads to Gag ubiquitination [23] and when directly fused to different retroviral Gags, Ub can functionally replace the L-domains [179,180]. Furthermore, Ub can be found in retroviral mature particles [181,182] and its depletion inhibits virus budding [22,23], while the recruitment of HECT Ub ligases, belonging to the Nedd4-like family, is clearly involved in retroviral particle release [23,183,184,185,186,187]. Interestingly, the PPXY type of L-domain interacts with the members of the Nedd4-like family of ubiquitin ligases by directly binding the WW domain characteristic of these cellular proteins [183]. Finally, the L-domains act independently from their position in the viral protein, frequently occur in combination and can be exchanged between unrelated viruses without losing their ability to mediate budding [188,189,190,191,192]. Overall, these features are suggestive of a role for the L-domains as docking sites for cellular factors belonging to a specific pathway involving Ub and exploited by retroviruses to efficiently execute budding. The role played by Ub in the endocytosis of transmembrane proteins suggested initially that the endocytic pathway could represent this cellular pathway [6]. Furthermore, some transmembrane proteins, such as the epithelial Na+ channel, were known to contain sequences perfectly overlapping retroviral L-domains which are involved in the process of endocytosis [193,194,195]. Moreover, it was demonstrated that the Ub residues involved in retroviral budding were indeed the residues involved in protein endocytosis [196]. However, the vesiculation process taking place during endocytosis is topologically opposite to the one occurring during viral budding. Indeed, while the budding of a virus happens from the cytosol toward the extracellular space and the factors that catalyze membrane fission must work from within the bud neck, during endocytosis the vesicles bud into the cytoplasm and membrane fission is driven by dynamin from outside of the bud neck. Thus, the cellular machinery involved in the formation of endocytic vesicles could not be the one exploited by viruses for their egress.

**Figure 3:**
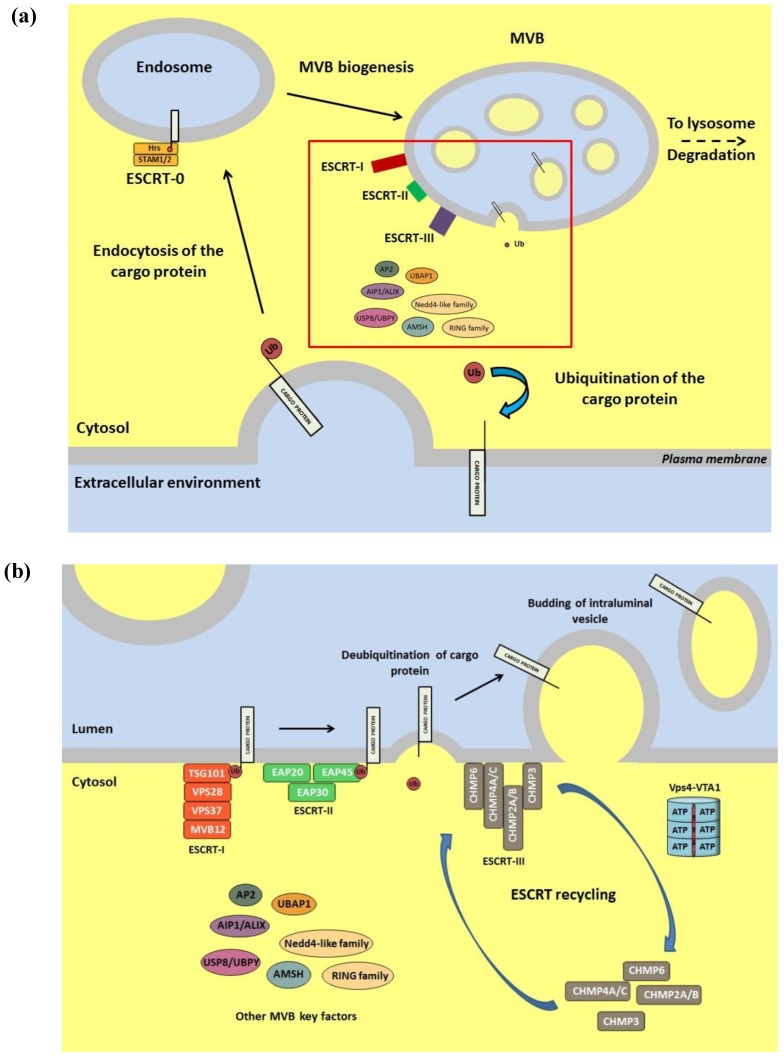
(**a**) Schematic representation of the MVB biogenesis pathway. An enlargement of the red squared part is shown in panel b; (**b**) Schematic representation of the vesiculation process leading to the formation of the MVB. The sequential recruitment of the ESCRT complexes to the MVB membrane is described along with the additional factors involved in the cargo protein delivery into the organelle lumen. The extracellular environment and its equivalents are colored in light blue, while the cytoplasmic environment is colored in yellow. Details on the ESCRT proteins and on the other MVB key factors can be found in several comprehensive reviews [7,169,173,201,202].

This apparent discrepancy between experimental data started to find a solution thanks to the seminal discoveries of the Carter’s laboratory [197] and of the Sundquist’s group [198], that identified in the cellular protein Tumor Suppressor Gene 101 (TSG101) the binding partner of the HIV1-1 PT/SAP motif, an interaction which is crucial for HIV budding. These initial findings, along with the work done beforehand and in parallel by cellular biologists, allowed to establish the connection between a cellular pathway, to which TSG101 belongs, and the HIV-1 egress from infected cells: the biogenesis pathway of an organelle of the endocytic pathway, the multivesicular bodies (MVB) [7]. Since then, more than 10 years of research have clarified that retroviruses, and in general most enveloped RNA and some DNA enveloped viruses, exploit MVBs during the latest steps of their replication cycle [173]. These organelles give reason of the connection between viral budding, ubiquitin, L-domains and the endocytosis of transmembrane proteins. Indeed, MVBs represent the organelles that eukaryotic cells have evolved to make the degradation of transmembrane proteins possible [7]. When such a protein needs to be removed from the plasma membrane, it is ubiquitinated and endocytosed on the surface of endosome. Then, a budding of vesicles from the membrane into the lumen of the endosome takes place, which allows the delivery of the trans-membrane protein into the lumen of the endosomes. This vesiculation event leads to the biogenesis of the MVB, which will eventually fuse with a lysosome resulting in the degradation of its cargo (Figure 3) [199,200]. It is clear that, if the transmembrane protein would not get access to the lumen of the endosome, thanks to the formation of the MVB, its degradation through the lysosome could not occur. Thus, the budding of vesicles from the endosomal membrane into the interior of the organelle (the biogenesis of the MVB) is the crucial step in this process. This vesiculation process, which takes place from the cytosol towards an environment that is equivalent to the extracellular space (the endosomal lumen), is now an event topologically identical to the budding of viruses from the plasma membrane. As a consequence, the connection between viral budding and MVBs biogenesis is functionally and physiologically sustainable. This vesicle budding step requires the sequential recruitment from the cytosol to the endosomal membrane of a highly conserved set of proteins, the ESCRT machinery [201] and different members of such a machinery, as TSG101 and AIP1/Alix are recruited by the viral L-domains to allow viral budding [56,173]. 

What is still apparently missing is the link between Ub, MVB and viral budding. Instead, several links do exist, with the most interesting one represented by the evidence that Ub plays a central role in the regulation of the MVB biogenesis pathway. Indeed, ubiquitination is necessary and sufficient to trigger the ESCRT-dependent endosomal sorting of membrane proteins and their degradation through the MVB/lysosomal pathway [202]. Moreover, precise and finely controlled cycles of ubiquitination and deubiquitination of target proteins and of specific components of the ESCRT machinery are essential for the function of the entire pathway of MVB biogenesis, till the vesicle budding [203,204,205]. Indeed, different upstream components of the ESCRT machinery with an essential role in viral budding, such as TSG101 and AIP1/Alix, are ubiquitinated and are able to bind Ub [206,207,208,209]. 

Overall, the aforementioned crucial role played by Ub in the control of the MVB biogenesis pathway by itself supports the strong evidence of an involvement of this cellular protein in viral budding. However, in addition or in parallel to this regulatory function, there is evidence that also direct ubiquitination of specific viral/cellular proteins might be important for the ESCRT-dependent viral budding. In particular, the importance of a direct Gag ubiquitination is supported by several studies, as reviewed by Votteler and Sundquist [173]. The working model foresees that ubiquitination of Gag would facilitate its interaction with the ESCRT machinery. In other words, ubiquitination of Gag would mimic ubiquitination of transmembrane proteins along the endocytic/MVB pathway, thus mediating the engagement of the ESCRT components. Under this respect, it has been shown that the binding of TSG101 to Gag has an increased affinity when Gag is linked to Ub [208,209]. Furthermore, the Bouamr’s group, by fusing the DUB domain of the HSV-1 UL36 to Gag and to specific ESCRT proteins, has recently reported evidence supporting a crucial role for Gag ubiquitination in HIV budding [210]. However, along with these data, other studies indicate a more complex scenario. For instance, it has been demonstrated that ubiquitination of Gag proteins can increase also in the absence of a functional L-domain and when budding is inhibited [211]. Moreover, the artificial targeting of Ub ligases to Gag leads to its ubiquitination, but not always enhances budding [187]. Finally, Zhadina and coworkers have nicely shown that Ub-dependent viral budding can take place without viral protein ubiquitination [212]. Thus, the question whether Gag ubiquitination is the result of a bystander effect or has a functional relevance is still open. Interestingly, it has been reported that the identity of the protein to which Ub is bound might not be the key element for viral budding [192]. What appears to be crucial is, instead, the presence of Ub at the site of budding, thus emphasizing its crucial role in the process. 

## 7. Conclusions

As very simple intracellular parasites, viruses rely on host cell factors and pathways to perform their life cycle and to establish a successful infection. The extensive use of Ub and UbL-mediated pathways by a number of unrelated viruses in different steps of their life cycle emphasizes the central importance of these cellular machinery in the cell physiology and, as a consequence, for viral replication and spreading. In this review, we have tried to give an overview of the complexity of the interplay between viruses and Ub/UbL-conjugating systems. While it appears evident that different aspects have been deeply analyzed and better understood, such as the role played by UPS in the context of the mechanisms evolved by viruses to control the cell cycle and to counteract innate immunity responses, more work needs to be done in order to clarify the functional relevance of Ub involvement in specific steps of viral replication, such as viral budding. Moreover, different emerging findings require further clarification both from the cellular and from the viral point of view, such as the role and the substrates of the newly characterized viral and cellular UbL proteins. Under this respect, the study of the viral connection with the Ub/UbL-conjugating machinery still represents one of the better examples of how viruses can serve as potent tools to dissect complex cellular processes and pathways. 
